# Normative positions towards COVID-19 contact-tracing apps: findings from a large-scale qualitative study in nine European countries

**DOI:** 10.1080/09581596.2021.1925634

**Published:** 2021-06-02

**Authors:** Federica Lucivero, Luca Marelli, Nora Hangel, Bettina Maria Zimmermann, Barbara Prainsack, Ilaria Galasso, Ruth Horn, Katharina Kieslich, Marjolein Lanzing, Elisa Lievevrouw, Fernandos Ongolly, Gabrielle Samuel, Tamar Sharon, Lotje Siffels, Emma Stendahl, Ine Van Hoyweghen

**Affiliations:** a Ethox and Wellcome Centre for Ethics and Humanities, University of Oxford, Oxford, UK; b Centre for Sociological Research, KU Leuven, Leuven, Belgium; c Department of Medical Biotechnology and Translational Medicine, University of Milan, Milan, Italy; d Department of Experimental Oncology, European Institute of Oncology IRCCS, Milan, Italy; e Institute of History and Ethics in Medicine, Technical University of Munich, Munchen, Germany; f Department of Political Science, University of Vienna, Veinna, Austria; g College of Business, University College Dublin, Dublin, Ireland; h Ethox Centre, University of Oxford, Oxford, UK; i Department of Philosophy, Universiteit van Amsterdam, Amsterdam, The Netherlands; j Department of Global Health and Social Medicine, King's College London andClinical Ethics and Law, University of Southampton, Southampton, UK; k Hub and Department of Ethics and Political Philosophy, Radboud University, Nijmegen, The Netherlands; l Philosophical Ethics and Political Philosophy, Radboud Universiteit, Nijmegen, Netherlands

**Keywords:** Contact-tracing apps, COVID-19, public perceptions, ethics, governance

## Abstract

Mobile applications for digital contact tracing have been developed and introduced around the world in response to the COVID-19 pandemic. Proposed as a tool to support ‘traditional’ forms of contact-tracing carried out to monitor contagion, these apps have triggered an intense debate with respect to their legal and ethical permissibility, social desirability and general feasibility. Based on a large-scale study including qualitative data from 349 interviews conducted in nine European countries (Austria, Belgium, France, Germany, Ireland, Italy, the Netherlands, German-speaking Switzerland, the United Kingdom), this paper shows that the binary framing often found in surveys and polls, which contrasts privacy concerns with the usefulness of these interventions for public health, does not capture the depth, breadth, and nuances of people’s positions towards COVID-19 contact-tracing apps. The paper provides a detailed account of how people arrive at certain normative positions by analysing the argumentative patterns, tropes and (moral) repertoires underpinning people’s perspectives on digital contact-tracing. Specifically, we identified a spectrum comprising five normative positions towards the use of COVID-19 contact-tracing apps: *opposition, scepticism of feasibility, pondered deliberation, resignation*, and *support*. We describe these stances and analyse the diversity of assumptions and values that underlie the normative orientations of our interviewees. We conclude by arguing that policy attempts to develop and implement these and other digital responses to the pandemic should move beyond the reiteration of binary framings, and instead cater to the variety of values, concerns and expectations that citizens voice in discussions about these types of public health interventions.

## Introduction

In March 2020,^1^ while many countries in Europe were experiencing lockdown measures to prevent the spread of COVID-19, technology developers and policy makers started exploring the possibility of deploying digital tools to contain the pandemic (Budd et al., 2020). In China and South Korea, digital tools based on collecting geolocation data were already being used to track people’s whereabouts, identify potential contagions due to proximity to subjects who had resulted positive to COVID-19 testing, and request those who were at risk of having contracted the disease to self-isolate. Soon, European countries developed and initiated mobile applications to support contact tracing, resorting to Bluetooth technology to identify proximity between devices, register symptomatic cases and send notification of exposure. Nonetheless, in spite of the great emphasis placed by European governments upon this technological intervention to contain the spread of the pandemic, the uptake of these apps in European countries – in terms of number of downloads and, more significantly, of active users – has been fairly limited, with figures consistently pointing to around a quarter of users among the whole population (see Supplementary Material). The stark gap between policy expectations on the use of these apps to address a public health emergency, on the one hand, and citizens’ modest uptake, on the other hand, calls for a critical analysis of this issue to further understand the underlying factors and implications.

There has been a strong cross-fertilisation between public and policy debates around these apps, both at the national and European level, revolving around their legal and ethical permissibility, social desirability as well as feasibility and efficacy. At the centre of such debates have been issues of privacy and data protection, such as anonymity and data storage, the use of Bluetooth versus GPS technology, decentralized versus centralized data gathering (e.g. Abeler et al., 2020; Schneble et al., 2020); but also issues of voluntary as opposed to mandatory use of contact tracing apps, in the absence of explicit or implicit legal or social pressure (e.g. Gasser et al., 2020; Parker et al., 2020), public-private partnerships in app-development, and open-source app design.

Scholars have articulated concerns around the need to provide justifications for privacy infringements and balance such infringements against the right to liberty (Parker et al., 2020) and/or public health needs (Martinez-Martin et al., 2020). Other scholars and practitioners have criticised a narrow reference to privacy as the mere protection of individual confidentiality, highlighting the fundamental democratic import of privacy (Blengino et al., 2020). In this spirit, some authors took aim at the predominant focus on privacy in public and political debates (e.g. Lanzing, 2020; Sharon, 2020; Siffels, 2020) and pointed to the more complex social and ethical landscape underpinning the development and use of these technologies, raising concerns for instance, as to the social injustice aggravated by the deployment of contact-tracing apps, benefiting mainly those who are already better off (e.g. Floridi, 2020). Issues of transparent governance and public trust have equally been posited as significant elements of concern for policy and public discourse (Bengio et al., 2020; Couch et al., 2020; Lucivero et al., 2020), on a par with matters of feasibility and efficiency of this proposed technological intervention (Braithwaite et al., 2020).

More broadly, these debates have occurred on the backdrop of ongoing policy discussions at the national and EU level on the need to advance a ‘European way’ to digital innovation (Albrecht, 2016; Thompson, 2018), predicated on values such as privacy, trustworthiness and transparency, and the parallel attempt to cast Europe’s role as a ‘global regulator’ (Erlanger & Satariano, 2020) in the digital domain. In addition, discussions around COVID-19 apps intersect with debates about the growing challenges posed by Big Tech corporations in the digital domain, and the means to devise and implement data governance mechanisms to ensure the ethical legitimacy and societal robustness of digital technologies (Prainsack, 2020; Sharon, 2020). For instance, it has been argued that the roll out of COVID-19 contact-tracing apps should be seen foremost as a form of ‘corporate contact-tracing,’ which is located at the intersection of surveillance (Zuboff, 2018) and disaster capitalism (Klein, 2007), and which epitomizes recent trends towards a greater influence of digital corporate platforms within publicly operated health systems (French et al., 2020).

Public and policy debates on digital contact-tracing apps have tended to unfold around a dichotomic framing of the issues at stake, whereby one option was assessed against the other on the basis of a perceived trade-off between infringement of privacy and the improvement of public health (Martinez-Martin et al., 2020). This framing carries two further – often implicit – assumptions. Firstly, it assumes that the potential pitfalls of digital contact-tracing mostly revolve around privacy issues, to be addressed through a set of national and international rules ensuring that this technology is not put to privacy-infringing uses. Secondly, it conveys a sense of inevitability, premised on a perceived linear progression from science to applications to ethics, whereby social and ethical considerations come into play to tame the potential negative consequences of this technology rather than informing its development from the onset.

Confronting these assumptions, this article departs from a linear perspective from science to ethics, and instead explores the views, expectations, and normative stances articulated by publics and citizens. Our work takes its cue from approaches in empirical ethics and pragmatist ethics (Keulartz et al., 2002; Willems & Pols, 2010) that highlight that lay people’s normative reasoning should be taken seriously when exploring ethical concerns. This is because their knowledge and beliefs rely on some form of expertise based on their experiences. These approaches draw on perspectives in Science and Technology Studies and the sociology of knowledge that critique the polarisation between expert and lay knowledge (Collins & Evans, 2002; Wynne, 2001), that have also inspired much literature in public health and sociology of medicine (Popay & Williams, 1996; Wilcox, 2010). In line with this literature, this study’s point of departure is that the debate on the ethical legitimacy of contact tracing apps cannot neglect or undermine citizens’ positions, concerns and beliefs and requires an in-depth articulation of the underlying arguments, values and reasoning. In this way, our study complements existing public surveys and polls that have been conducted to gauge public views on COVID-19 apps and that provide only initial and limited insights on people’s opinions of digital contact-tracing apps.

Against this backdrop, our aim is to provide an in-depth account of the distinct *normative positions* that can be distilled from the views on digital contact-tracing technology expressed by citizens in nine European countries during 349 in-depth qualitative interviews. Rather than merely ‘polling’ respondents’ preferences and views towards the app, our goal is to provide a detailed and nuanced account of *how* people *arrive* at elaborating a certain normative position: Which arguments do they articulate in support of their views and reasoning? Which values are invoked? What is the moral vocabulary that people mobilise? In order to achieve this goal, we paid specific attention to the argumentative patterns, tropes and (moral) repertoires^2^ underpinning people’s perspectives, so as to identify distinctive normative positions possessing an overall internal coherence, which we outline and discuss in the main corpus of the article.

## Methodology

This work is part of the Solidarity in Times of a Pandemic project (SolPan), which uses in-depth interviews and qualitative data analysis methods to investigate the views and practices of people from nine countries in Europe in dealing with the COVID-19 pandemic crisis (see Supplementary Material). The SolPan consortium developed collaboratively a semi-structured interview guide, which included questions on people’s views on the planned deployment of digital tools to support tracking and tracing. Recruitment took place via e-mail lists, social media, and personal contacts. Each of the nine country teams carried out between 22 and 80 in-depth qualitative interviews via online platforms or via telephone in all nine countries between 6 April and 6 May 2020. In total, the topic of digital contact tracing arose in 282 out of 349 interviews.

Using a codebook developed collaboratively,^3^ we analysed the empirical data first within each country team in the language of origin and then shared memos in English summarising the most important themes and insights. In this analysis, we found important common patterns across countries, and a few specificities that were obviously related to specific events such as earlier app rollouts. For this reason, rather than analysing the data in a systematically comparative way, we organised the data along the positions that emerged from an analysis of the themes articulated in all countries. Within each theme, we describe and illustrate different nuances of articulation.^4^

## Findings

**Figure 1.**
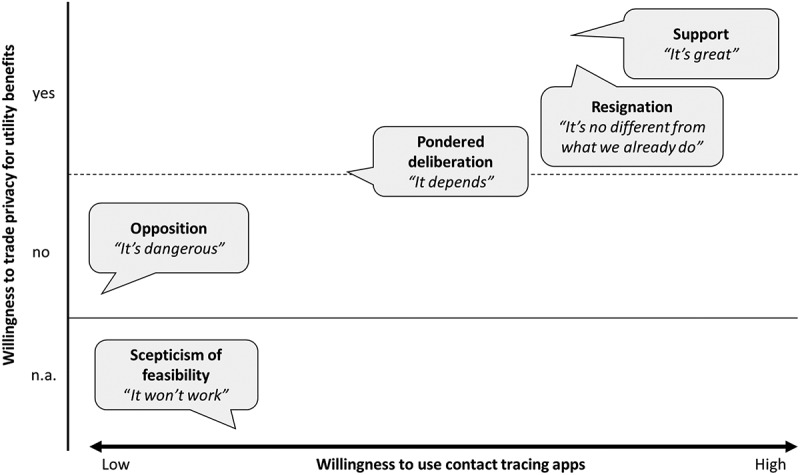
Spectrum of normative positions. (n.a. = not applicable).

We identified a spectrum of five different normative positions towards the use of COVID-19 apps (Figure 1). In what follows we outline each of these positions, highlighting the kernel of the position expressed, the major argumentative patterns and tropes employed, the type of normative arguments being articulated, the values at stake, and whether COVID-19 apps are perceived as introducing moral disruption with respect to well-established digital practices. It is important to highlight that inasmuch as the focus of this work is on charting the *views* and *arguments* in relation to this proposed technological solution in-depth, rather than merely polling people’s preferences, the stances traced here are not mutually exclusive, meaning that a single respondent may articulate different normative stances in the same interview.

### The ‘opposition’ position

It’s the door open to a whole bunch of threats to our liberty. (FRCP04)^5^

The first position expresses *aversion* towards the use of COVID-19 apps based on strong concerns about governments or other powerful actors reinforcing already pervasive surveillance structures in our hyper-connected digital societies. Respondents acknowledge that widespread exposure to tracking practices (through e.g. geo-localization, social media use, web searches) routinely occur, and, within this context, perceive the use of COVID-19 apps as posing an increased and unacceptable threat in terms of loss of privacy and heightened control.

Arguments articulated by respondents holding this position tend to follow a *deontological type of reasoning*, foregrounding the protection of some rights perceived as inalienable (e.g. individual autonomy and freedom), irrespective of whether or not a specific practice is likely to cause actual harm. They are often framed as *slippery slope arguments* pointing to the risks of ‘function creep’, namely the re-use of data and information outside the scope for which they had been originally collected, as well as drifts towards unaccountable and undemocratic regimes of surveillance. Typically, this stance is expressed through the use of strongly evocative tropes, conveying a sense of fear and danger through references to ‘surveillance’ (UKGS01; UKAP01, DEAS11, DEAB05), ‘Big Brother’ (UKSMO2; UKSR04; UKSH02), ‘witch hunts’ (DEBZ07), ‘Chinese practices/state’ (DEAB06, NLML03), ‘living like in a prison’ (NLLS07), or people being ‘programmed’ (ATEW09, NLMP04).

In broad terms, respondents perceived COVID-19 apps as raising additional concerns with respect to already existing digital practices and technologies, inasmuch as they are seen as enabling a (i) *deeper* or (ii) *more coercive* form of control, and (iii) as possibly entailing forms of *social stigmatization*.

- (i) Some respondents frame contact-tracing apps as a step too far in a context where people already give away vast troves of personal data in exchange for services of different sorts (e.g. digital financial transactions), while exposing themselves to potential future harms deriving from the use of such data. The fear being expressed is that of heightened function creep, perceived by respondents as a defining tenet of our societies, which risks being further exacerbated by the large-scale deployment of COVID-19 apps. The (often implicit) reasoning underpinning these concerns contrasts well-established social practices predicated on intensive data-gathering (like using credit and debit cards) with the uptake of digital contact-tracing technologies: Whilst respondents may be reluctantly prepared to face the potentially negative consequences deriving from the former (since it may not be possible or desirable to give up on them), they are much less inclined to do so in the context of COVID-19 apps, as exemplified by this quote from an Austrian respondent:

We’re already giving away so many things and we don’t know how that could be used against us in the future - not that I’m totally crazy there now and I’m not paying with a cash machine, and I have paranoia or something, but yes. You should still be a bit careful and I believe that the state has far too much power over you. (ATWS01)

- (ii) Another reason for concern is the feeling of ‘imposition from above’ that surrounds COVID-19 apps. Unlike other digital practices based on the voluntary engagement of people, mostly as consumers of digital services, the deployment of COVID-19 apps by public authorities is perceived – in spite of the strong emphasis placed on voluntariness in policy debates – as a coercive practice jeopardizing the autonomy and freedoms of people:

I mean it’s now true that we are tracked everywhere we go because most people have Facebook, most people have … eh … Instagram and so anyway, if they want to track us, they can track us. But this thing is more … it seems more imposed, so the imposition would bother me. (ITIG13)

- (iii) Thirdly, concerns around COVID-19 apps are also heightened by the fear of stigmatization that may derive from the use of contact tracing technologies within the broader public health toolkit:

And it’s like a stigmatization in principle, if I exaggerate just a little bit, it’s like the Star of David back then [that Jews had to wear under Nazi rule]. Because then I also knew who had passed me by. Who he is and who he isn’t. And when that [information] lights up electronically on the app, that’s the same now with Corona. And next there will be other infectious diseases or anything else, where I get a warning every time a person passes by. And I just don’t think that’s right. (ATLS03)

Through either of these argumentative routes, opposition to COVID-19 apps boils down to the perceived excessive risks posed by contact-tracing technologies to people’s privacy – which is portrayed not only as an individual good, but also as a proxy for a higher democratic value. For many of our respondents, COVID-19 apps are seen as epitomizing a controlling state that carries out the thorough monitoring of its citizens and ‘*knows exactly’* (DEAB06) their whereabouts at a very granular level. The worry of ‘*being watched all the time’* (IESA04) is a major reason for opposing COVID-19 apps, also to avoid the envisaged drift towards a situation akin to that experience in highly technologically surveilled and undemocratic societies (with China being often identified as a case in point):

For me, it’s the door open to a whole bunch of threats to our liberty. Clearly, I think that we are really in an emergency situation, which is the perfect breeding ground for dictatorships and for a dictatorship of security, in the form of protectionism. That’s what I am saying. Using protectionist arguments to protect us, we are starting to use cameras and new technologies to … No, that really raises questions. I’m very concerned about this kind of thing. When we see what is happening in China with regard to the social contract, etc., I find it very worrisome. I find it very worrisome (FRCP04)

### The ‘scepticism of feasibility’ position

I wonder if that would work. (CHBZ25)

The second position, also on the critical side of the spectrum, is characterised by a focus on the *practical feasibility* of this technological intervention, which is accompanied by the *scepticism* that the digital contact-tracing system will work as expected. Respondents articulate an eminently *pragmatic* type of reasoning, expressing their *scepticism* towards the effectiveness of COVID-19 apps and their successful deployment to control the spread of contagions. Therefore, holders of this position do not engage in full-fledged normative discussions around the reasons for either supporting or opposing the adoption of this technology: the question of whether COVID-19 apps will promote individual or the public good, or in fact raise unacceptable concerns, is dismissed (or left pending) on pragmatic grounds, as no digital contact-tracing system is expected to be successfully set up in the very first place.

Anyway, it’s not clear how we’re going to use this yet. We’re going to find out, so we’ve got a person, we’ve just met a person who may be susceptible to being infected with it, […] no, who actually has the disease, and then what do we do with it? Besides this tracking, what do we do with it? We’re going to get a phone call, but then what? So that’s what I’m wary of, yes. (FRMG05)

Scepticism towards the effective deployment of COVID-19 apps is expressed in several different ways, by referring to the population as (i) ‘unready’ or (ii) ‘unwilling’ to use this technology properly, on the one hand, and (iii) to the lack of adequate governance and infrastructural capacity by public authorities to support the implementation of the app, on the other hand.

(i) First, respondents question the *societal readiness* for COVID apps due to a *lack of digital literacy and digital uptake*, especially by the older population:

Italy is not a nation like China where everyone has a smartphone. The elderly, or other disadvantaged people who have no possibility to buy a smartphone, this is a big limit […] The problem is that, in my opinion, having this app on the phone … I don’t think it will change the spread of the virus. I think it won’t help. At least, here in Italy it won’t help until Italy will be a super modernized and technological country (ITLM04)

Such arguments are given weight on the basis of considerations pertaining to personal habits (‘*I’ve noticed that if I go to the store, I leave my phone in the car, so that I’m not bothered all the time. I leave the phone at home often too*’ – NLLS09), inter-generational differences (‘*I’m from a different generation […] For me, it’s kind of difficult media-wise, I must say. I wouldn’t know how to handle it*’ – CHBZ18), family experience (‘*My husband would […] definitely lose out because he always forgets his mobile phone anyway*’ UKGS05), or interactions in professional contexts (‘*in my work I see that people do not know how to operate digital technologies and switch on a Bluetooth on their phone*’ – ITFL03).

(ii) Second, respondents question people’s *willingness* to use the app and abide by the authorities’ recommendations. This view is framed as a broad judgment about human behaviour and the compliance towards norms that are often non-strictly binding or difficult to enforce, and whose effectiveness therefore hinges on the personal responsibility of citizens.

I honestly don’t know what to make of it. In itself, it wouldn’t be such a bad idea. But from what I’ve heard, it would require everyone to be honest about their state of health and so on. And that’s the point where I wonder if that would work … whether really everyone is honest or willing to give out their data. (CHBZ25)

(iii) Third, scepticism towards the effectiveness of digital contact-tracing systems is also addressed towards the *governance and organisational structure* that is required to enable a successful implementation of such systems. In particular, people express a lack of confidence in the capacity of existing public health infrastructures to accommodate digital-contact tracing technologies, most notably in light of the challenges faced by health authorities in keeping track of infected people or scaling up testing through non-digitised public health surveillance systems:

I think it is hardly applicable because they are not doing enough testing and then people cannot know whether they are positive or not. Then, either everyone is tested and then everyone downloads it and everyone controls, or it is useless as there is the problem of asymptomatic people. (ITFL04)

### The ‘pondered deliberation’ position

And then what’s more important: is it the public good or your personal data privacy? I think it is a very complicated debate. (IEFO04)

The third stance, characterized by the *process* through which people articulate their normative reasoning as much as its outcome, is centred on the *pondered deliberation* of the different motives for either supporting or opposing COVID-19 apps. The argumentation is often articulated in the guise of a risk/benefit assessment: that is an *explicit appraisal* of the different reasons for either supporting or opposing COVID-19 apps. Substantively, this position is typically expressed by pitting concerns towards digital monitoring and privacy against the potential benefits deriving from the use of digital contact-tracing systems. The latter, as we outline below, are perceived as revolving in particular around a number of public or personal goods (public health, economic recovery, personal freedom) that COVID-19 apps are expected to help attain. The argument is thus of consequentialist nature, focusing on the potential *usefulness* of the technology. In most cases, this pondered deliberation around the pros and cons of COVID-19 apps tips the balance of the argument in favour of this technology, as the identified benefits are believed – in the end – to outweigh the privacy concerns.

At a more fine-grained level, we can identify two dominant argumentative patterns underpinning the views of people engaging in such deliberation: (i) pitting the personal vs the public good; (ii) reasoning around the limits of the present state of exception.

(i) As we have just noted, the concerns of people holding this stance largely revolve around possible infringements to *personal* privacy, which is therefore conceived of here as a good that mostly impinges on the individual level, rather than a fundamental value of relevance for society as a whole; conversely, the benefits associated with the use of COVID-19 apps are presented, by most, as the achievement of some form of *public* (rather than individual) good. Consequently, through this framing that puts (a lesser) personal benefit in opposition to what is considered (a higher) public benefit, most people engaging in this deliberative reasoning end up supporting the adoption of COVID-19 apps, despite the reservations they may have:

It’s a complicated one I think because obviously you have ethical considerations, privacy considerations, data privacy considerations. And then what’s more important: is it the public good or your personal data privacy? I think it is a very complicated debate, I suppose, where there is a huge ethical dimension that needs to be teased out and needs to be part of the debate, if we were to go this avenue. Personally, perhaps, I would be open to it, just in terms of if it helps the greater good, I would be perhaps willing to compromise on my privacy in that sense. Yeah, so something that needs careful consideration, but I think I could be personally persuaded if it’s done right. (IEFO04)

When it comes to articulate what the ‘public good’ amounts to, people recurrently point to the following aspects. In the most basic and recurrent sense, COVID-19 apps were perceived as functional to public health efforts, so as to ‘*break the chain of transmission of the virus*’ (FRRH02), ‘*control the virus better*’ (UKAF02), ‘*prevent a second wave*’ of infections (CHBZ10), while also relieving healthcare systems that have been put under severe stress by the pandemic. All the while, COVID-19 apps are further framed as ‘*the lesser evil than another lockdown*’ (ATEW01; ATKR09), inasmuch as they are expected to ‘*ease a return to the normal*’ (NLLS01, DEAS10) and bring about benefits for the economy, while also improving the psycho-social wellbeing of people severely affected by the pandemic. In terms largely coincident to those employed in the following quote, people expressed the view that

blocking a country for two months is really harmful, both from the economic point of view and from the psychological point of view of people, because it is unfortunately felt every day of people who maybe try or succeed in committing suicide, and it is really absurd. (ITIG04).

It should be noted, however, that opposition of personal versus public benefit was not always so clearly articulated; in balancing benefits and drawbacks, some respondents advanced a distinct line of argument centred on the additional *personal* benefits that the use of the app could bring forth, stating that they prefer to trade their privacy in order to gain back some valued personal goods, such as the freedom of movement.

I have problems with individual freedom when I’m prevented from circulating. By preventing me from going where I want […]. It’s been two months, it’s going to be two months. […] That’s enough. (FRRH02)

Equally, it should be stressed that, even where people acknowledge their preference to their preference to cede their privacy in order to gain more liberty of movement, this is not always unconditional, as they may want to retain some control over the amount of privacy they give up: ‘*afterwards, who’s going to keep all the data? Is it going to be sold to laboratories?*’ (FRTS01).

(ii) Another strand of argumentation within the deliberative attitude revolves around a type of reasoning that foregrounds the exceptional character of the current pandemic. The public health and social toll of the crisis was perceived as a strong-enough reason to endorse the use of COVID-19 apps within the context of the present situation (e.g. ‘*in this context, if it just helps to contain [the pandemic], it’s different*’ (CHBZ06); ‘*I’m ok […] for the time being. Afterwards, of course it’s something else*’, (FRTS02). However, similarly to the first position on the spectrum, concerns are raised that normative changes introduced on the basis of what is perceived as a present state of exception can then lead to a normalization of otherwise socially disruptive and ethically contestable measures. Again here, people envisage a slippery slope, whereby the present adoption of what are perceived as largely privacy-invasive practices will lend legitimacy to future applications that are considered unethical outside of the present situation and in absence of appropriate safeguards.

In some cases, recent precedents, such as digital monitoring introduced in the aftermath of terrorist attacks in the US, France and the UK, were invoked in support of such concerns. People express fears that digital technologies that could be suited to ensure effective *public health* surveillance could become a means for *population* surveillance, once the pandemic is over.

I understand, of course, that this can be used to do contact tracing and it can potentially contain how the virus is spreading. And I can already see the usefulness of that. It’s just, my problem is-, and I’m struggling with that too. How does a crisis justify us to intervene in basic human rights? And I think the problem is that if there is no pushback, you might end up in a situation. I mean, think how it was just after the terrorist attacks in London and in the US, when the NSAs of the world all of a sudden were very active. And then nobody really knows what information they have? And that’s not how it should be like in a democracy. So I think that citizens should take great care that some things are very difficult to return to or to make undone. (DEAS10)

### The ‘resignation’ position

We already let everything and everyone trace us. So it’s okay. (ITLM03)

A fourth position expressed by respondents is one of *resignation*, predicated on the acknowledgment that we have already accepted being tracked in many spheres of our lives (for example, by large tech companies). Such (sometimes reluctant) acceptance of technology-based tracking justifies the acceptance of contact-tracing apps as a matter of coherence with other spheres of life. Although it starts from the same premise of the *adverse* position (‘we are continuously monitored’), it reaches an opposite conclusion (‘we can be monitored by the app as well’). Within this position, two slightly different views can be disentangled: a reluctant and a more overtly resigned one.

- (i) On the one hand, while acknowledging the reality of technology-driven monitoring and the need to accept the app as a matter of coherence, some respondents express their uneasiness with the situation, together with an awareness of having little control over it.

Realistically, if they wanted to, they could probably track us already anyway … . I’d prefer if they didn’t keep track of me. But, like I said, they can probably already do it. (UKSM04)

- (ii) On the other hand, a second stance with more overtly resigned undertones posits that since technology-driven monitoring and tracking is already happening and we have become accustomed to it, these apps are in line with current practices and do not introduce much disruption. On the contrary, they have the added value of being ‘more visible’. Hence, contrary to those who express reasons for concern in light of the feeling of ‘imposition from above’ that surrounds COVID-19 apps (see above), for some of those expressing a resigned stance the public visibility (and thus potentially heightened accountability) of this technology represents a positive aspect:

I assume that this happens [tracking] anyway under the radar. I just think it’s the first time that they declare it […]. I think it’s just a bit of an irony that they’re asking permission now and they’ve never asked permission before. (UKSM03)

### The ‘support’ position

That’s no problem at all. (DEBZ06)

The final stance is one of outright *support* of the proposed technological solution. While purportedly representing a ‘*small thing*’ requiring little to no effort from individuals, the app is viewed here as an ‘*effective*’ means to tackle the pandemic crisis:

I really believe in these apps because I think that, if they are handled consistently, they could really reveal important information to the authorities to develop specific measures or to react to the course of the pandemic. (DEBZ03)

Similar to the other positions (bar the ‘scepticism of feasibility’ one) traced above, reference to *privacy issues* often represents the starting point for the unfolding of this normative position. In fact, contrary to some other positions on the spectrum (e.g. the one of opposition), privacy concerns are invoked here precisely to *downplay* their perceived relevance in the assessment of apps in the current situation. This act of downplaying privacy concerns occurs through either of the following argumentative patterns.

i) For one thing, some people argued not to have had ‘*any concerns about privacy [even] before [the pandemic]*’ (CHBZ24), with some referring to privacy as an ‘*overrated*’, ‘*exaggerated*’ notion (CHBZ24). Therefore, all the more in the present situation, privacy cannot represent a major concern to hinder the adoption of COVID-19 apps. Secondly, in an implicit recognition of the privacy implications of this technology, some others argue that endorsement of the app stems from having themselves ‘*nothing to worry about*’ (FRCP02) and wondering what others *‘do that is so secret that they aren’t allowed to know?’* (NLLS12). An interviewee articulating this position went on to note, ‘*a convicted felon*’ may actually have ‘*a different idea*’ on this point (ITFL03).

ii) Other respondents, although concerned about privacy, acknowledge that ‘*sometimes you have got to sacrifice your privacy for greater good’* (IEIG03). They take an overtly positive stance towards the perceived intensive data gathering activities of the app, and frame the use of this technology in terms of outright ‘*data donation*’: they recognize that the app is inherently bound to make extensive use of their personal data, and are eager to actively provide their own data to counter the effects of the pandemic (‘*That’s no problem at all*’) (DEBZ06).

## Discussion and conclusion

As our findings show (see Table 1 for an overview), views about apps were justified by referring to different values and through a number of normative arguments. A consistent element in the spectrum of stances that we have mapped is that the respondents tended to frame their thoughts in terms of trade-offs that people are willing to accept (or not) in order to use the apps. If the *pondered deliberation, resignation* and *supporting* stances are held by people who are willing (for different reasons and with increasing levels of enthusiasm) to allow access to personal data in view of a greater good, the *opposition* stance is not willing to accept this trade-off. The focus on trade-offs reflects how the discussion on the ethics of contact tracing apps has been framed by public media, policy discussions, and in some scholarly debate. It is reasonable to believe that the wider public and media debate has played a role in shaping people’s views. At the same time, our analysis enriches the debate on COVID-19 apps because it shows that not all engagements with apps are framed in this dichotomous way, and that positions falling on both sides of the ‘divide’ are often articulated by the same person. The *sceptical* argument assumes that it is impossible to choose between seemingly competing value systems. Instead, issues of feasibility are brought forward to raise broader concerns about societal and organisational readiness.

**Table 1.** Overview of different positions

Position	Description *(in a survey we would only learn what is after the comma)*	Type of argument	Values at stake	(Moral) exceptionalism of COVID apps
Opposition	*I’m concerned, I will not use it*	Fear of surveillance, distrust towards State, resistance against a societal trend (deontological/rights-based argument)	Autonomy, individual freedom (individuals not used as means); democracy	yes > fear of slippery slope
Scepticism of feasibility	*It will not work; I will not use it*	Scepticism towards societal and governance readiness	n/a	n/a
Pondered deliberation	*It’s a complicated issue, but in the end, it may be worth using it*	Trade-offs between privacy vs other goods (consequentialist argument)	Autonomy, individual freedom (privacy); public health; economy; societal well-being; individual freedom (of movement)	yes > fear of slippery slope
Resignation	*It’s not different from what we already do, we should use it*	Coherence with ongoing practices	Individual freedom; public health	No
Support	*It’s great, I will use it*	Rejection of the value of privacy; believe in the usefulness of app for containment	Effectiveness	No

Our analysis also enriches this discussion insofar as it disentangles the trade-off framing, moving beyond the simple dichotomy between individual interest (in terms of privacy) and the public good, to describe a full spectrum of positions that focus on a number of aspects and values, consequently elaborating different moral approaches to the use of the app. For example, in the analysis of the *opposition* stance, people’s resistance towards a dangerous societal trend protects not only individual freedom against an over-controlling state, but a broader value of democracy, individual and collective liberty. This resonates with scholarship that argues for privacy as a social, not just an individual value, and that views privacy as necessary for achieving other values, such as democracy, freedom, psychological wellbeing, and creating and maintaining social relationships (Roessler & Mokrosinska, 2015; Solove, 2008). Furthermore, when apps are perceived as an alternative to lockdowns, being free to move seems more valuable than privacy. Different things seemed to matter to our respondents (economy, public health, freedom of movement, individual health, democracy, a sense of ‘normalcy’). Hence, articulating the variety of values, concerns and expectations in people’s views of these apps is a first step to engaging in a meaningful discussion and understanding the reasons for uptake of these types of interventions.

In addition, our findings allow us to chart citizens’ attitudes towards the *governance* of digital technologies and some of their underpinning *political dynamics* with fine-grained empirical resolution. In broad terms, many interviews show that people do not have confidence in the readiness of current infrastructures for the delivery and governance of digital technologies. In spite of the considerable emphasis placed within Europe on advancing a value-friendly ‘European way’ to digital innovation, doubts persist as to the reach and effectiveness of such efforts (e.g. Marelli et al., 2020; McMahon et al., 2020). In line with these arguments, our findings indicate that people – irrespectively of the views they express on issues of privacy and digital tracking – widely conceive of governments as unable to tame the dataveillance practices that are routinely carried out by corporate actors, which can be interpreted as the perception of overall significant limitations of current data governance models.

Additionally, COVID-19 tracing apps were perceived as laying the basis for additional thorough surveillance structures, to be actively carried out by the state apparatus. More to the point, the view being expressed is that, once such technologies are woven into the social fabric justified by the urgency of exceptional circumstances, they could be endowed with normative and social legitimation that propel their further use for socially disruptive and ethically illegitimate purposes. In the same vein, political leadership is portrayed as taking advantage of the COVID-19 crisis to pursue other and broader political goals.

These concerns seem to reveal two distinct – yet mutually supporting – underlying argumentative patterns. On the one hand, digital technologies are perceived as quintessentially privacy-intrusive, and therefore as embedding as well as enabling a politics of surveillance. Concerns here seem to refer to the *inherent affordances* provided by such technologies (cf. Winner, 1980), which enable large-scale data harvesting, tracking of people and can be thus brought to the task of enacting widespread regimes of control. On the other hand, people also displayed concern for the *political dynamics* that underpin the deployment of digital technologies, and which seem to carry equal weight for the potential ‘threats to our liberties’. Notably, interviewees seem to point to, and show awareness of, a consistent *modus operandi* in the current political climate, whereby governments across the whole political spectrum increasingly seem to justify unpopular measures bound to erode fundamental democratic and socio-economic rights – in the digital domain but also beyond – on the basis of ‘states of exception’ that are perpetuated once the ‘crisis’ of the moment is over (Agamben, 2005; Bagnai, 2014; French et al., 2020; Klein, 2007; Mirowski, 2013). Pervasive digital surveillance enacted in the aftermath of terrorist attacks in Western countries such as the UK, France and the US is only but perhaps the most conspicuous example of this.

Against this backdrop, our findings cut across longstanding debates around the current pitfalls of data governance in Europe and beyond, and point to the heightened relevance of improving the underlying politics of digital technologies, and the need to come up with socially robust governance mechanisms – if we are to weave such technologies, effectively and democratically, into the social fabric.

## Supplementary Material

Supplemental Material

## Notes

1. This publication has been made possible by the joint work of the members of the SolPan research commons.

2. We define these notions along the line of the classic work by Swierstra and Rip (2007) on the ethics of new and emerging technologies. Thus, ‘trope’ can be understood as ‘a recurring motif or argument that is supposed to have particular force’; ‘pattern’ can be understood as ‘two or more ethical arguments that hang together in the sense that they provoke each other into existence’. In turn, tropes and argumentative patterns may become ‘repertoires’ that ‘are available in late-modern societies, both as a framing of how actors view issues and expect others to view them, and as a kind of toolkit that can be drawn upon in concrete debates’ (Swierstra & Rip, 2007, p. 4).

3. Codebook available at https://papers.ssrn.com/sol3/papers.cfm?Abstract_id=3776127.

4. A comparative approach on the same topic has instead been pursued in the work of Zimmermann et al. (2021).

5. The pseudonyms employed have been devised in the following way: the first two letters indicate the initial of the country where the interview was conducted (e.g. FR = France), and are followed by the initials of the person who conducted the interview and a number indicating the progressive order of the interview (e.g. 04 is the fourth interview conducted by CP in France).
