# Supplementary material for: Normative positions towards COVID-19 contact-tracing apps: findings from a large-scale qualitative study in nine European countries
Source: Crit Public Health. 2021 Jun 2;32(1):5–18. doi: 10.1080/09581596.2021.1925634 (PMC10292825; doi:10.1080/09581596.2021.1925634)
Supplement: Supplemental Material [file CCPH_A_1925634_SM3999.pdf]

Supplementary material

Data on uptake of these apps in European countries

Based on the publicly available information, the disaggregated figures on the downloads of COVID-19 in the nine countries mapped in our study (which is described in the methodology section), as in February 2021, are the following: 8-10% (active users, Oct. 2020) in Austria (https://viecer.univie.ac.at/corona-blog/corona-blog-beitraege/corona-dynamiken9/); 19,1% in Belgium (Dec. 2020, https://www.vrt.be/vrtnws/nl/2020/12/21/corona-app-amper-gebruikt/); 30% in Germany (https://www.rki.de/DE/Content/InfAZ/N/Neuartiges_Coronavirus/WarnApp/Archiv_Kennzahlen/Kennzahlen_11022021.pdf?__blob=publicationFile);19% (downloads) in France (https://www.service-public.fr/particuliers/actualites/A14683); 26,5% (active users) in Ireland (https://www.irishtimes.com/news/health/covid-tracker-app-issues-close-contact-alerts-to-20-000-people-in-six-months-1.4459373); 17,1% in Italy (https://www.immuni.italia.it/dashboard.html); 35% (downloads), 20,7% (active users) in Switzerland (https://www.experimental.bfs.admin.ch/expstat/en/home/innovative-methods/swisscovid-app-monitoring.html); 26% in The Netherlands (https://coronamelder.nl); 36% (downloads) in the UK (https://faq.covid19.nhs.uk/article/KA-01368/en-us?parentid=CAT-01052&rootid=)These data have not been independently verified, they are approximate and taken at different points in time

Solidarity and Pandemic

The project was initiated by Barbara Prainsack and Katharina Kieslich in mid-March 2020, has led to the establishment of a European multi-national consortium (the SolPan consortium) including Austria, Belgium, France, Germany, Ireland, Italy, the Netherlands, German-speaking Switzerland, the United Kingdom. A non-European arm of the study, the SolPan+ consortium, has been subsequently set out and it currently comprises 15 Latin American countries.

Methodology

In several countries, the first phase of interviews within the SolPan project coincided with the first "hot phase" of debates about COVID-19 apps. We therefore decided to ask our participants their views on technology-assisted tracking and contact tracing. Due to differences between countries in the development and roll-out of the app respectively as well as national media coverage of COVID-19 apps, in some countries the question focused specifically on these apps, while in others it was formulated as a general question about the use of digital technologies to help contain the spread of the virus. We invited participants to share their views on the apps, and we asked those who had experiences with the apps, or opinions on their use, follow-up questions to ascertain the reasons behind their views and positions. Of all the respondents who voiced opinions on the app (N= 282), only four – all of whom resided in Austria – had personal experience with using them.

Analysis proceeded in a two-pronged manner. We first prepared an overview about country-specific policies and the trajectory of development of the respective apps. We wrote these overviews on the basis of document-based research into public discourse and policy on tracing apps. These overview texts described the particular circumstances of the countries regarding policies, current spread of COVID-19, lockdown measures, progress in development regarding contact tracing apps, as well as legal issues and societal debates in all nine countries. Secondly, we analysed the interview transcripts, using qualitative research software Atlas.ti (version 8), where we labelled all relevant text passages with the code "tracking" or related subcodes including ethical and feasibility concerns towards the app.

The results of the data analysis in all country teams were discussed in weekly to biweekly meetings. In a next step, each country team filled in and translated the respective quotes for the themes in a joint table. From there, we conceptualised the themes into five categories as presented in this paper. The procedure was again a collaborative process about overlaps and redundancies among the identified themes across the country teams. The two first authors took the lead in producing the analysis and outline of this paper and all contributing researchers were invited to contribute and comment on the collaboratively resulting texts and contribute to the discussion.
